# Supplementary material for: Burst of gyrification in the human brain after birth
Source: Commun Biol. 2025 May 26;8:805. doi: 10.1038/s42003-025-08155-z (PMC12106832; doi:10.1038/s42003-025-08155-z)
Supplement: Supplementary file 1 — Supplementary Information [file 42003_2025_8155_MOESM1_ESM.pdf]

Supplementary

Supplementary Figures:

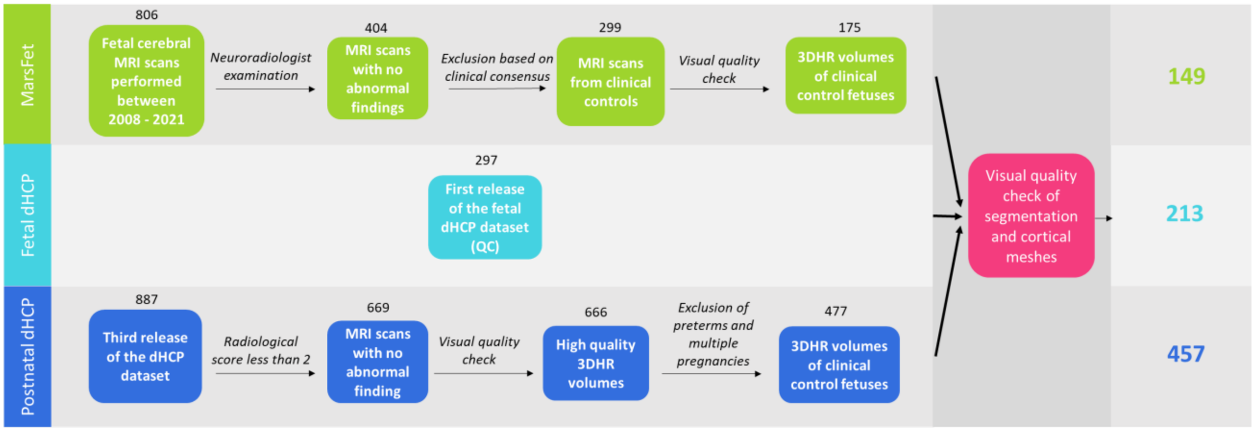

Supplementary Figure 1: Flow chart of data selection.

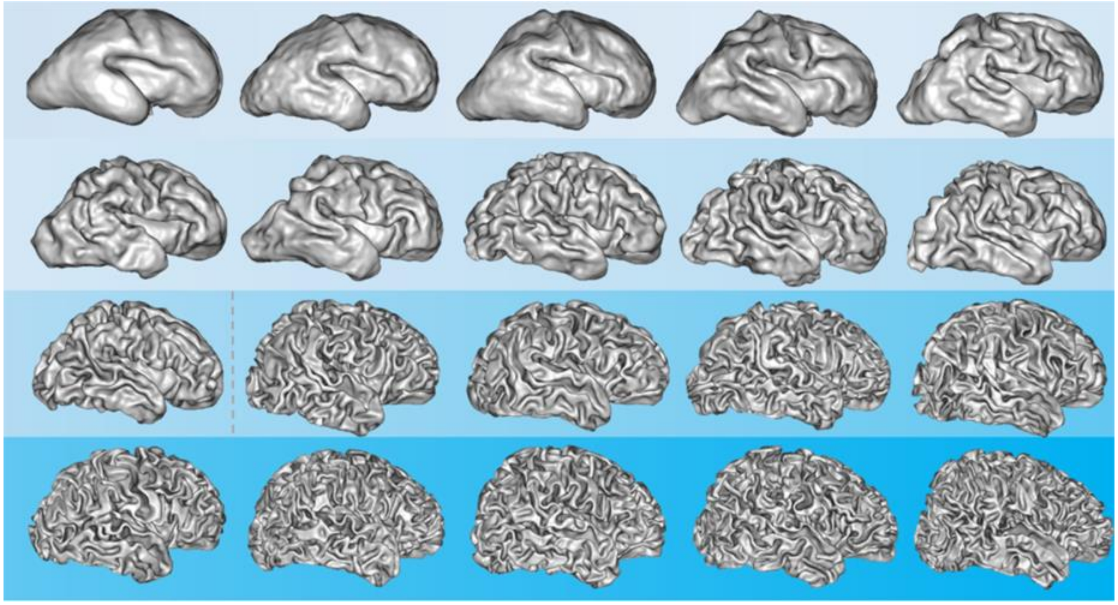

Supplementary Figure 2: Examples of cortical surfaces obtained using our pipeline. We show one brain per each week post-conception from 26 to 45 weeks (left to right, along each row). Note the increasing folding with age. The dotted gray line represents the transition from a fetal to a postnatal environment.

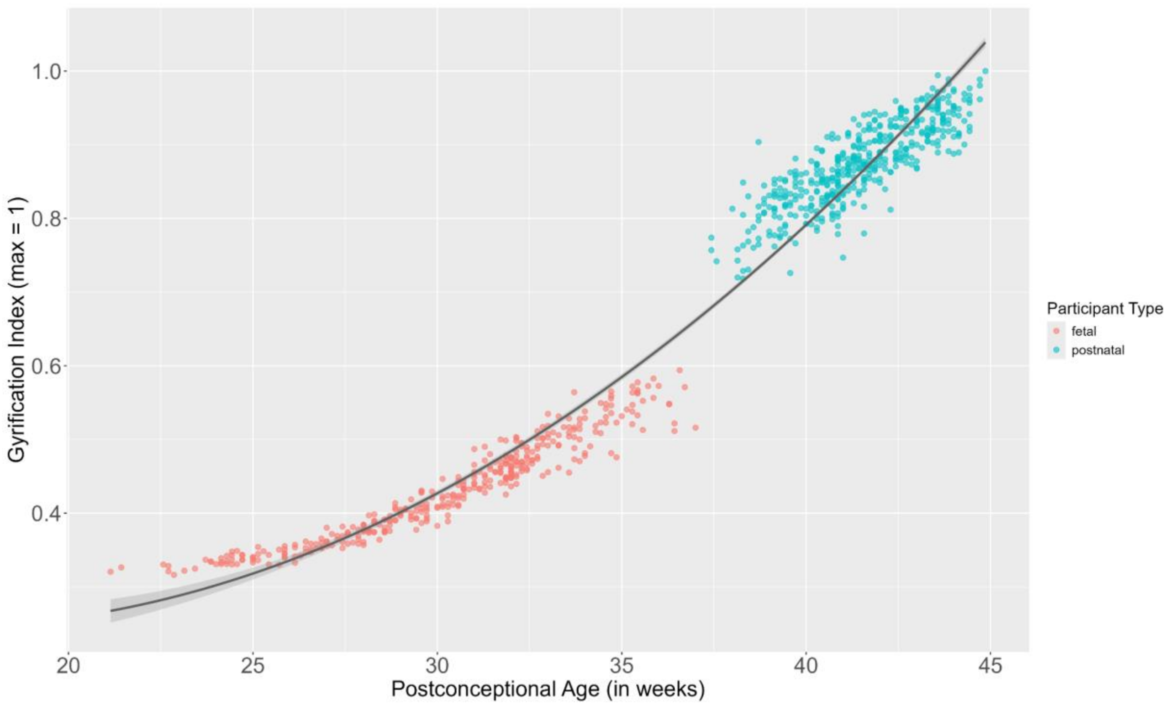

Supplementary Figure 3: Quadratic regression model fit on gyrification as a function of postconceptional age in weeks (*$p < 0.001$*, *$R^2 = 0.96$; residual standard error = 0.04)*. A noticeable jump around the time of birth can be seen, prompting a subsequent regression discontinuity analysis.

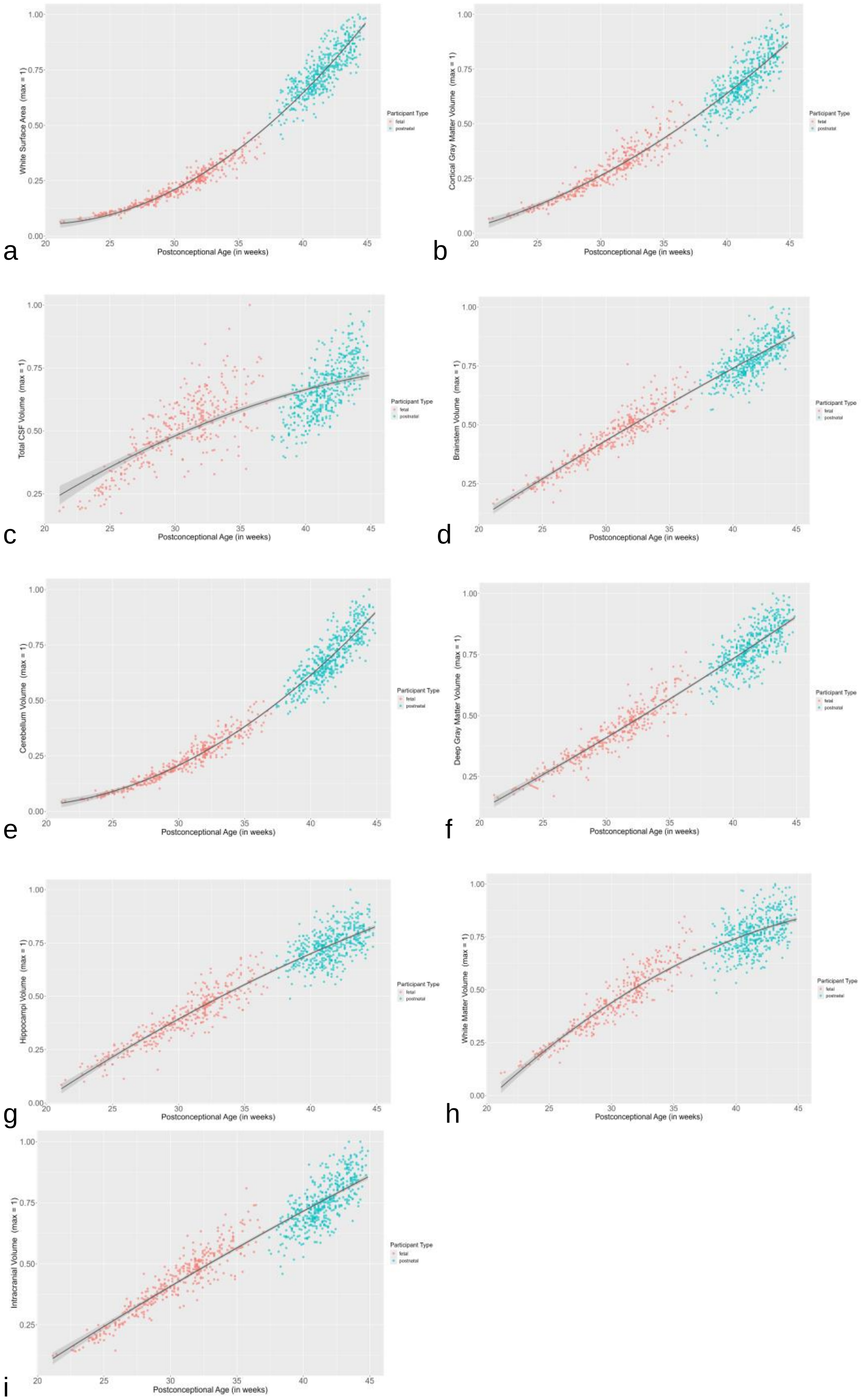

Supplementary Figure 4: To see if any other features in the brain exhibit obvious changes at birth, we also computed the quadratic models on cortical gray matter, subcortical gray matter, white matter, hippocampal, brainstem, cerebellar, cerebrospinal fluid (CSF), and overall intracranial volumes, as well as on surface area.

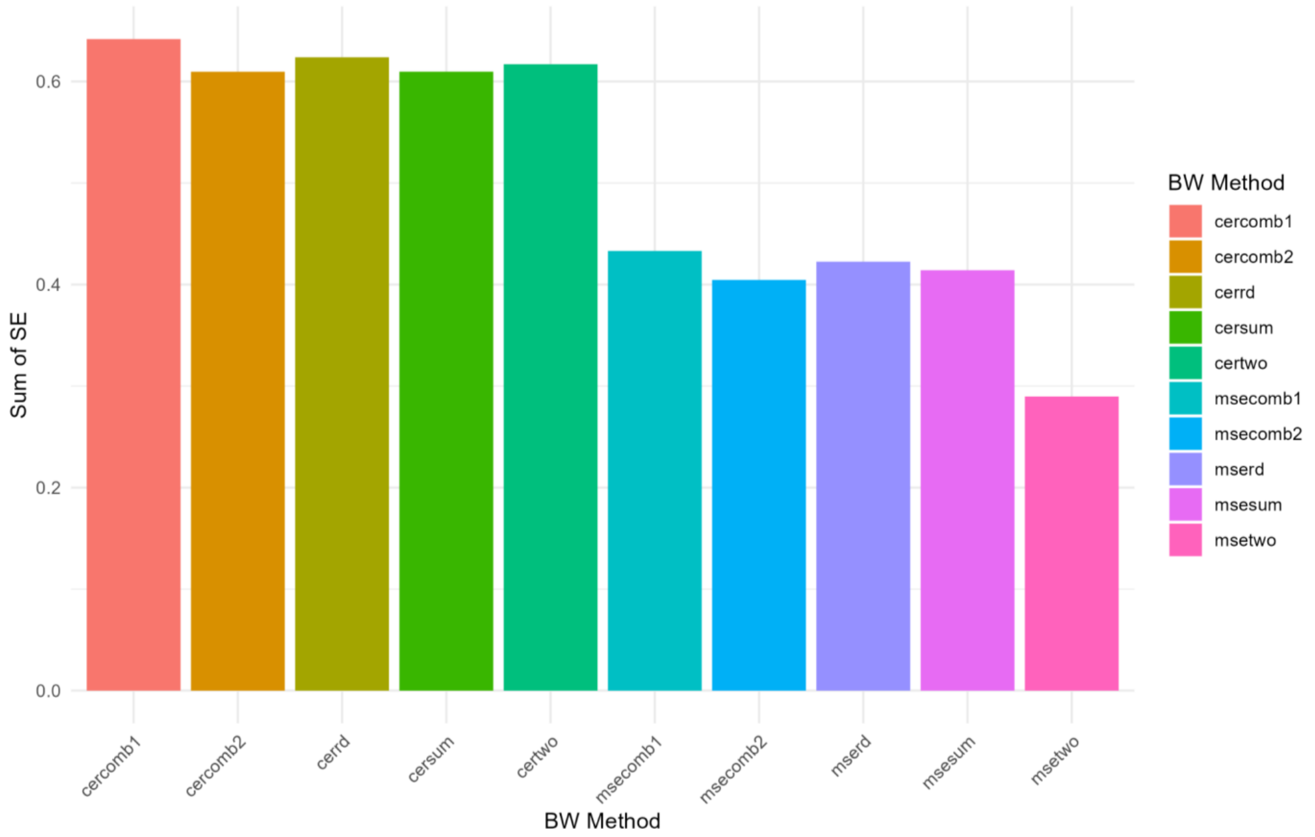

Supplementary Figure 5: Plot showing the sum of the standard error (SE) for each bandwidth (BW) selection method. The sum is based on iterating a regression discontinuity model over several cortical features, changing the bandwidth selection method and kernel type each time. Then the output standard error of each iteration was summed per bandwidth. Method “*msetwo*” showed the least error across model iterations.

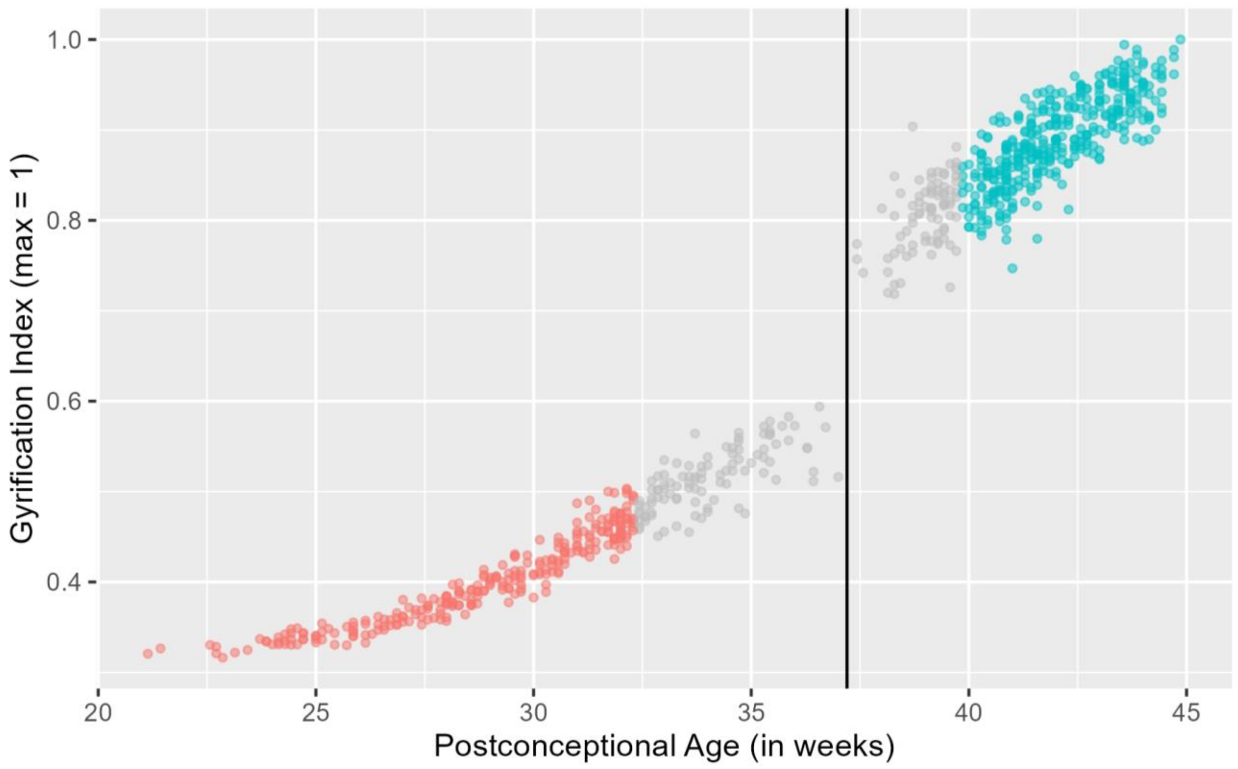

Supplementary Figure 6: The bandwidth selection method (*msetwo*) determined the optimal bandwidth lengths to be included in the RDD analysis from either side of the cut-off (a length of 4.8 units below and 2.6 units above the cut-off). This is represented by the gray shaded points.

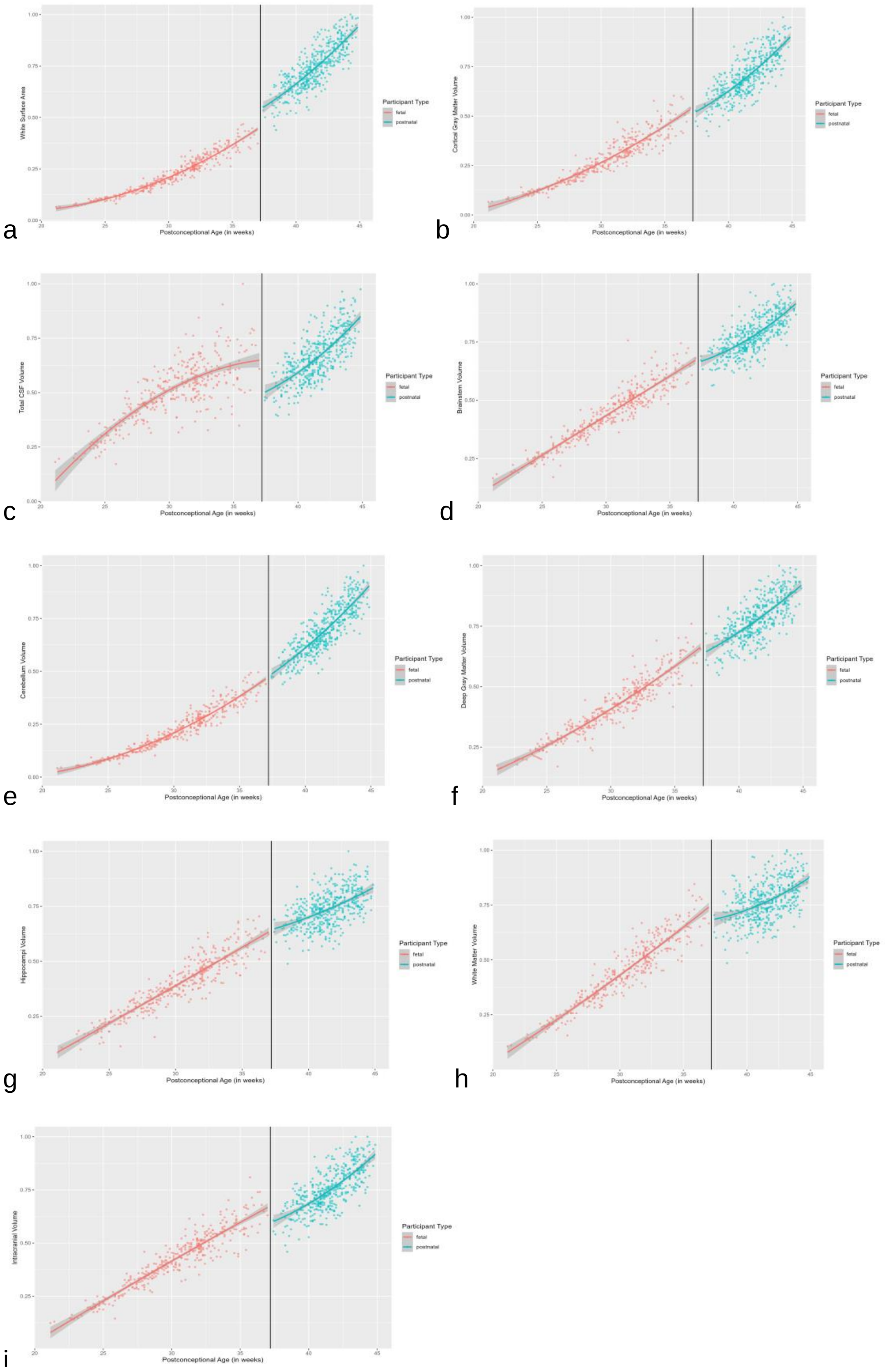

Supplementary Figure 7: To further enforce our statistically significant result of a discontinuity at birth within the neurodevelopmental trajectory of gyrification obtained by applying an RDD, we applied this same analysis to several other cortical features to rule out the possibility that our result is due to methodological inconsistencies. Furthermore, the RDD analysis statistically characterizes patterns visualized at birth in all features. All features were standardized to a maximum of 1 to facilitate interpretation.

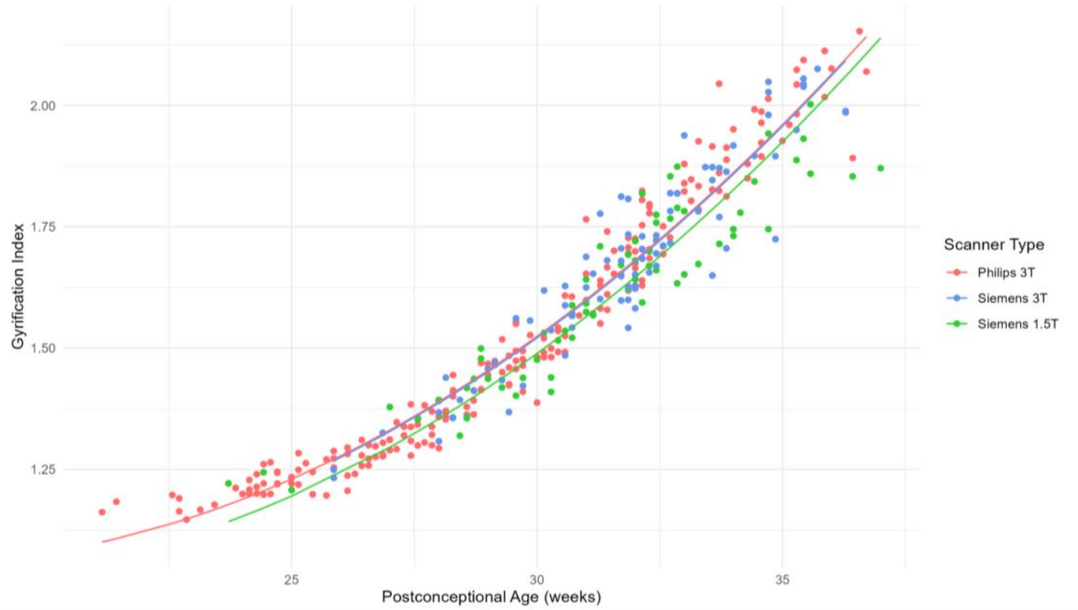

Supplementary Figure 8: Gyrification trajectory as a function of age for fetal participants across three scanner types. Two different Siemens scanners (1.5T and 3T) were used for the fetal MarsFet cohort, while a Philips scanner (3T) was used for the fetal dHCP cohort. An ANOVA indicates that there was a significant effect of scanner type within the fetal sample (*$p = 4.29x10^{-4}$*). For the MarsFet 3T scanner versus the MarsFet 1.5T scanner - *effect size = 0.24, p = 0.015.* For the dHCP 3T scanner versus the MarsFet 1.5T scanner - *effect size = 0.09, $p = 8.35x10^{-5}$*. No significant difference between the two 3T scanners was reported (*effect size = 0.15, p = 0.255*).

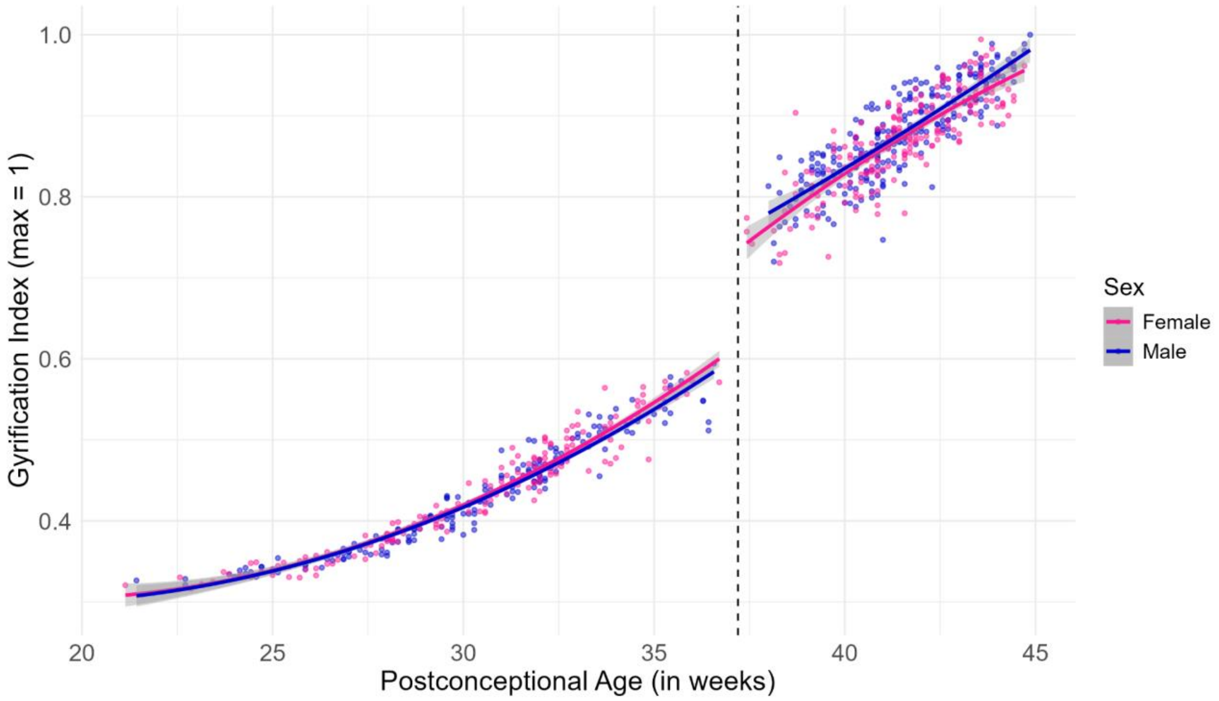

Supplementary Figure 9: Plot showing the neurodevelopmental trajectory for each sex. Visualization and statistical analyses (results section 2.5.3) confirm that this jump in discontinuity at birth is not affected by sex (*effect size = 7.58, $p = 3.28x10^{-14}$*).

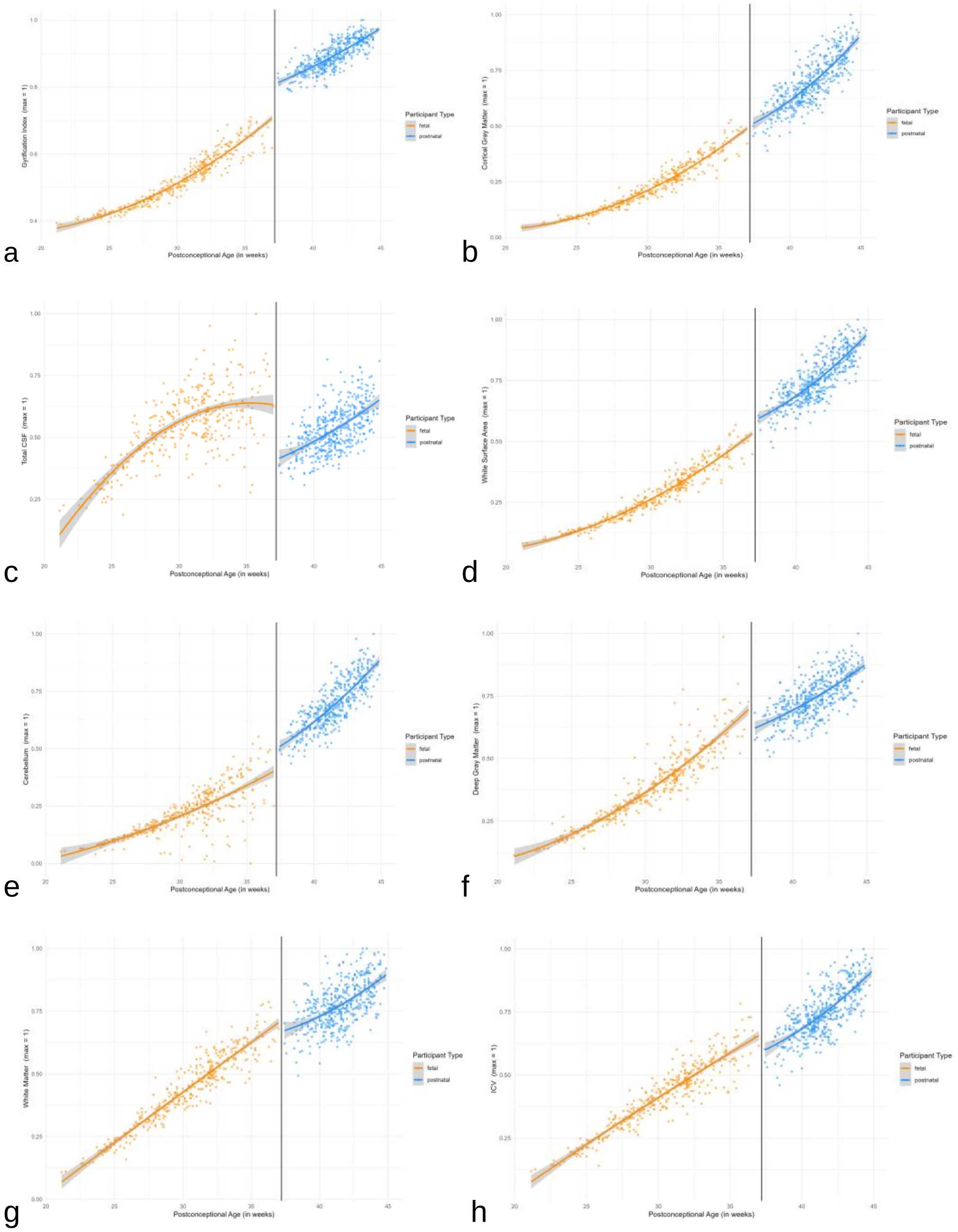

Supplementary Figure 10: Plots illustrating RDDs applied on synonymous features segmented by BOUNTI including gyrification, cortical gray matter volume, surface area, total CSF volume, cerebellum volume, deep gray matter volume, white matter volume and overall intracranial volume.

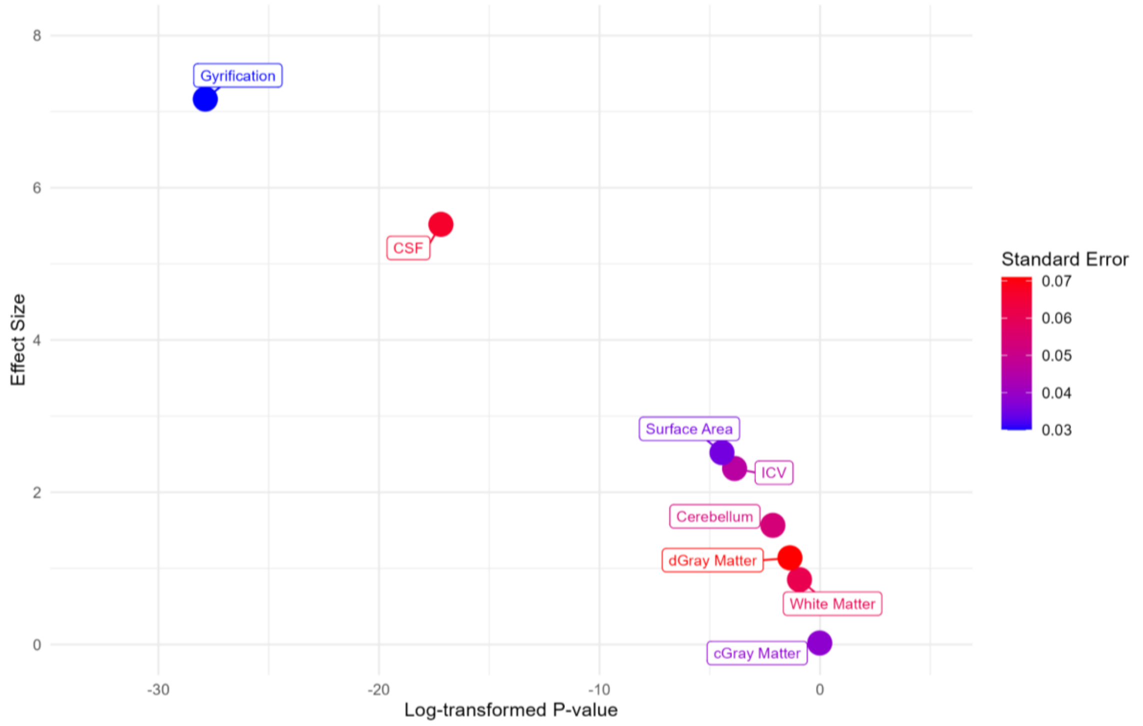

Supplementary Figure 11: Effect size as a function of log-transformed p-value for the regression discontinuity analysis in each **BOUNTI-segmented** feature (a higher effect size indicates a larger discontinuity at our cut-off, i.e., birth). Gyrification still shows a strikingly high effect size compared to other features. Remaining features have comparable statistical outputs as for the nnUNet-segmented data.

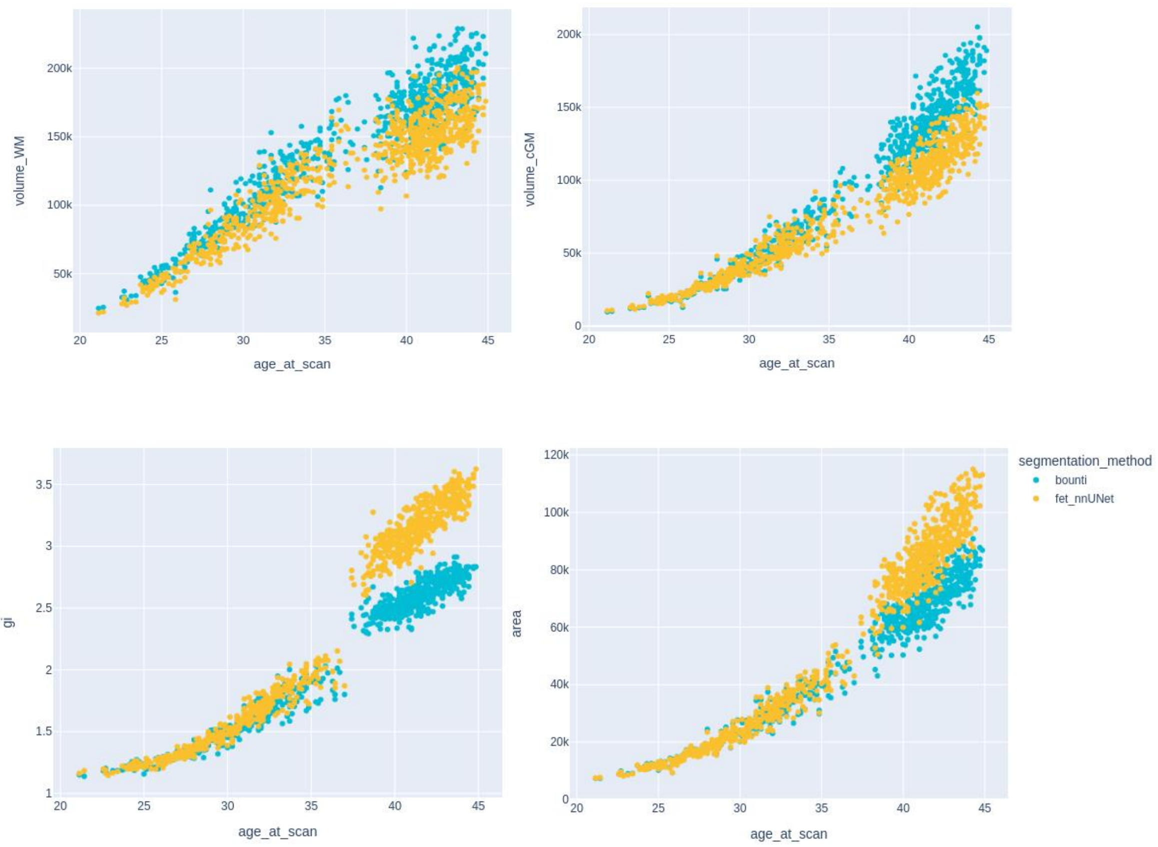

Supplementary Figure 12: Scatter plots of the most important features as a function of the age illustrating the comparison between the BOUNTI and nnUNet segmentations in our cohort. For all features, the measures are very consistent for fetuses, but differences are noticeable for postnatal participants. BOUNTI results in higher cGM volume and lower WM volume compared to nnUNet. The surface area is higher for nnUNet than for BOUNTI, which induces a higher gyrification measure for nnUNet compared to BOUNTI. Note however that the discontinuity at birth is still visible for gyrification while the other features evolve continuously across the perinatal period (bottom left). cGM denotes cortical gray matter, WM denotes white matter, and gi denotes gyrification.

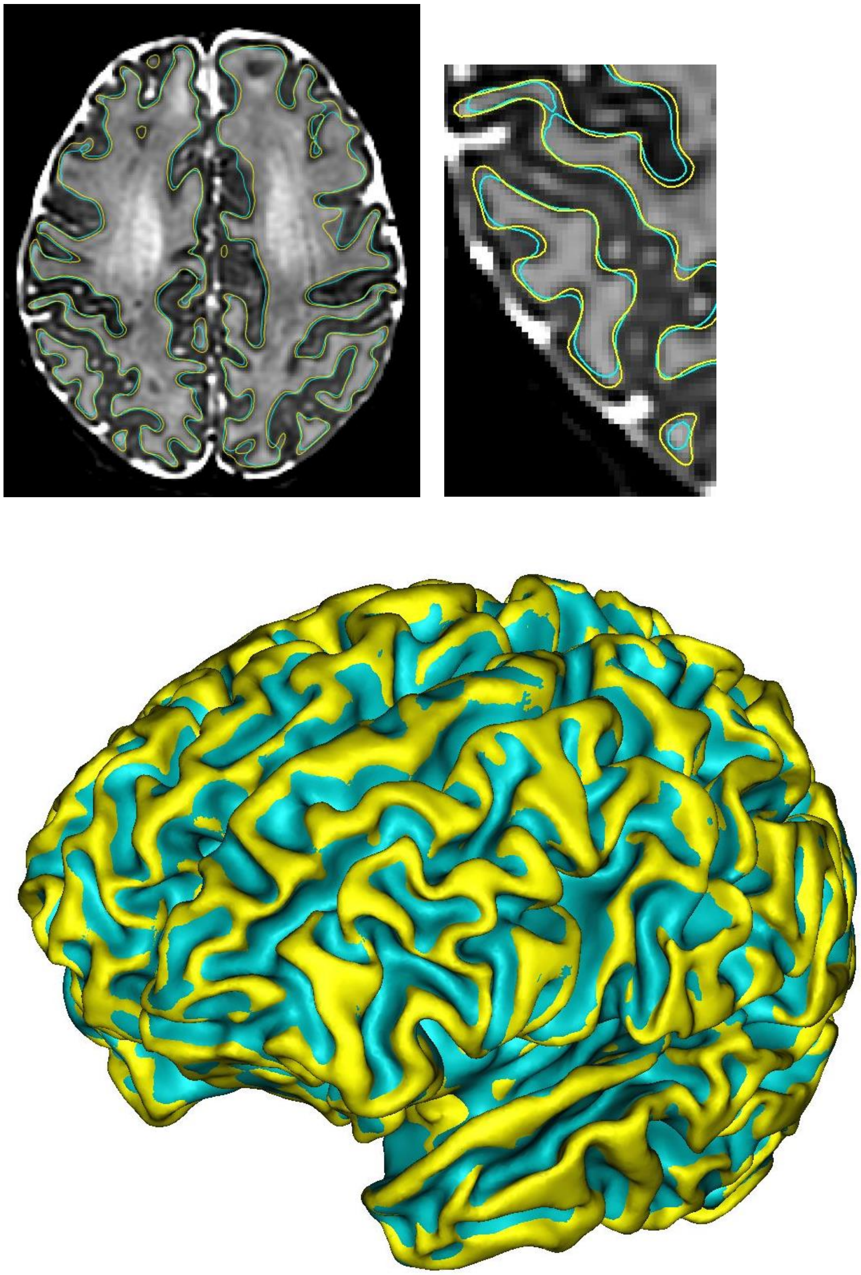

Supplementary Figure 13: Top - Illustration of the difference between the white matter surface obtained using BOUNTI (cyan) and nnUNet (yellow) for a representative individual. The segmentation of the cortical gray matter volume with BOUNTI is too smooth and does not accurately follow the interface between gray and white matter, in particular at the bottom of sulci. As a result, the surface from nnUNet (yellow) has a higher surface area and is more convoluted, with a higher gyrification index. Bottom - As shown by superimposing both surfaces on an anatomical scan, the surface from nnUNet is more accurate than from BOUNTI.

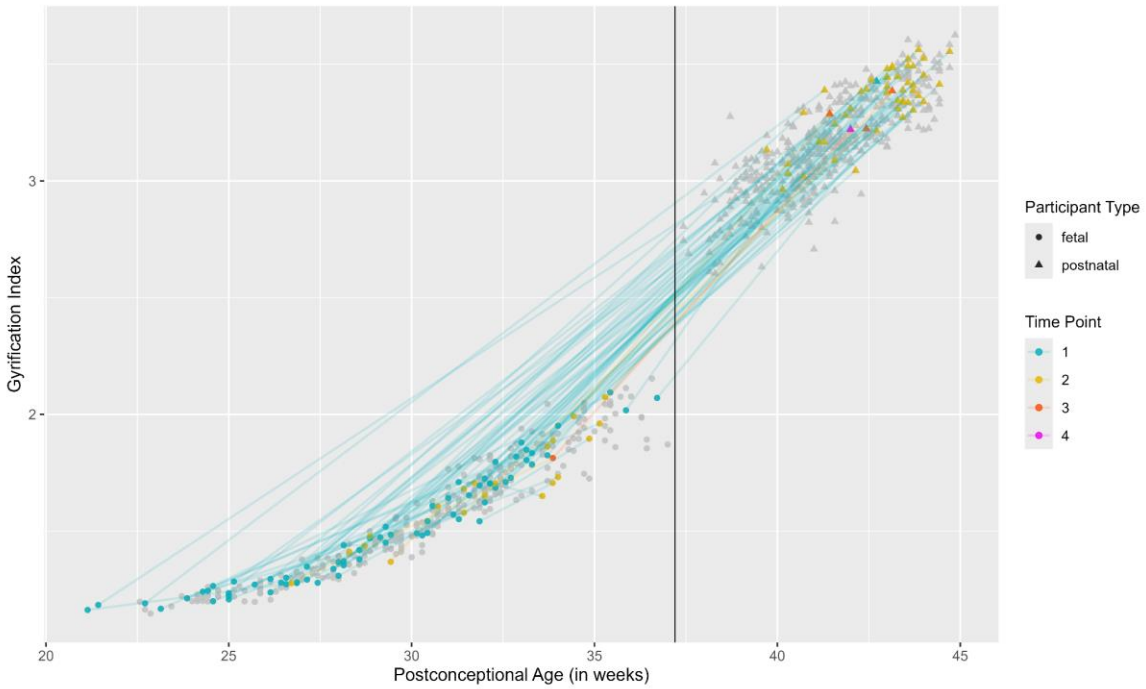

Supplementary Figure 14: Plot of all subjects having more than one timepoint (coloured according to number of timepoints, from one to four) superimposed onto remaining subjects (gray points) that do not have longitudinal data. Specifically, blue points represent the first timepoint in subjects with longitudinal data, yellow points represent the second timepoint, orange points represent the third timepoint and pink points represent the fourth timepoint, if available. As shown, not many subjects have more than two timepoints, and even less have timepoints before and after birth. Furthermore, as shown, even if all subjects had a few timepoints, it would be nowhere near the resolution required to conduct a longitudinal study that would demonstrate the important biological shift in gyrification at birth.

Supplementary tables:

Bandwidth Sensitivity Testing	Effect Size	P-value	95% Confidence Interval
**Bandwidths Generated by Model (*4.8, 2.6*)**	**7.48**	**6.44×10^{-14}**	**0.15, 0.25**
Doubled Bandwidths (*9.6, 5.2*)	10.40	2.42×10^{-25}	0.14, 0.21
Halved Bandwidths (*2.4, 1.3*)	4.22	2.42×10^{-5}	0.13, 0.37

Supplementary Table 1: To enforce the robustness of the bandwidth lengths computed by the model on either side of the cut-off, the RDD was run again with doubled and then halved bandwidth lengths. As shown on the table, the effect size remained large and the p-values remained significant even after changing the number of subjects included in the RDD model from before and after the birth cut-off. This enforces the strength of our results.

Variable	Coefficient	Standard Error	Left Bandwidth	Right Bandwidth	p value	Effect Size
Gyrification	0.214	0.029	3.919	1.810	6.26E-12	7.164
Cortical Gray Matter	0.00075	0.038	5.758	2.298	0.984	0.019
Surface Area	0.088	0.035	4.755	1.679	0.088	2.520
Total CSF	0.367	0.066	5.292	2.206	2.73E-07	5.518
Cerebellum	0.083	0.053	7.082	2.090	0.117	1.564
Deep Gray Matter	0.080	0.071	6.746	2.057	0.254	1.138
White Matter	0.052	0.061	6.820	2.146	0.393	0.854
ICV	0.107	0.046	6.429	2.033	0.160	2.312

Supplementary Table 2: Table summarizing RDD output statistics of the BOUNTI-segmented data. All significant p-values were Bonferroni-corrected.

Supplementary note 1: Comparison between BOUNTI and nnUNet segmentation approaches

BOUNTI is a deep learning-based fetal brain segmentation tool introduced to accommodate the lack of robust brain segmentation methods for fetal MRI[2]. The model was trained on 360 MRI datasets with different acquisition parameters. The accuracy of the method was assessed by computing developmental trajectories of 390 brain images from three cohorts acquired with different acquisition protocols, and showed robust performance across the 21 - 36 wPC range and with different acquisition protocols[2]. In the present study, we apply BOUNTI on datasets covering a larger age range. In particular, the application of BOUNTI to postnatal MRI data from the dHCP cohort (37 - 45 wPC) goes beyond the recommendations of the authors. Our observations confirm that the accuracy of the segmentation resulting from BOUNTI is very good for fetuses but limited for postnatal participants. As shown in Supplementary Figure 12, the segmentation from BOUNTI and nnUNet is highly consistent for fetuses. The measures, however, are quite different for postnatal participants. Careful visual assessment confirmed that nnUNet is more accurate than BOUNTI on postnatal participants, which is expected since data from postnatal participants were used to train the nnUNet model but not the BOUNTI model that was trained only on younger, smoother brains. More specifically, BOUNTI results in a much smoother segmentation of the cortical gray matter that does not reach the bottom of sulci and the crown of gyri (Supplementary Figure 13). As a consequence, the white surface is too smooth which results in an underestimate of the surface area and gyrification. Note that despite the limited accuracy of BOUNTI on postnatal participants, we still observe a discontinuity in gyrification but not for the other features (Supplementary Figure 10, Supplementary Table 2), which is consistent with our RDD analyses reported above in Figure 4 and Supplementary Figure 7.

Supplementary note 2: Mapping of longitudinal data along the gyrification trajectory

The current study was conducted on cross-sectional data. Running such an investigation on longitudinal data would be beneficial because the evolution with age could be estimated at the individual level. Note however, that it would not solve challenges brought on from imaging participants in very different conditions (fetal versus postnatal differences in acquisition). Indeed, even if a subject is scanned both before and after birth, the differences in acquisition setting (i.e., head coil after birth only) will induce potential confounding factors that cannot be avoided, such that controlling for potential effects using sensitivity analyses will still be required. Furthermore, estimating the trajectory of gyrification at the individual level would require a substantial amount of time points per subject (with strong constraints on the timing since we want to characterize the impact of birth), which has never been achieved due to challenges involved with obtaining MRI data in such sensitive populations. To offer a complete perspective on this study, we investigated the possibility of implementing longitudinal statistical models on our dataset. Within our pool of subjects, we have n = 69 participants with two timepoints, n = 4 with three timepoints, and n = 1 with 4 timepoints. Specifically, only n = 52 participants have at least one timepoint the in the fetal period and one in the postnatal period. Supplementary Figure 14 illustrates the number of timepoints and their age along the trajectory of subjects with longitudinal data available in our cohort. In addition to the limits induced by the low sample size, majority of acquisitions are not sufficiently close to birth precluding the relevance of a longitudinal design in the current study. Extending the research protocol implemented in[3] to cover the postnatal period would be a relevant option to be implemented in future studies.

Supplementary note 3: RDD model parameter selection

A regression discontinuity model was fit on gyrification as a function of postconceptional age using a polynomial order of 2 with a triangular kernel. In order to decide the bandwidth length, which is a critical factor in the model, the model was iterated over all available bandwidth selection methods. Model outputs were scrutinized so that the bandwidth selection method with the lowest standard error was finally chosen, namely *msetwo* – corresponding to a method that uses two different mean squared error (MSE) optimal bandwidth selectors, one each for below and above the cut-off (Supplementary Figure 5). The *msetwo* bandwidth selection method determines the appropriate bandwidth length for our data using data-driven approaches[1]. Computing a unique bandwidth for either side is appropriate since the fetal population necessitates a longer bandwidth (*bandwidth length = 4.8*) due to less participants around the cut-off compared to the postnatal population (*bandwidth length = 2.6*)(Supplementary Figure 6). The robustness of our results was confirmed by re-running our model with half the bandwidth length, and again with double to bandwidth length, in order to be certain that output statistics remain stable and are not sensitive to model alterations (Supplementary Table 1).

Supplementary References

1. Imbens, G. & Kalyanaraman, K. Optimal Bandwidth Choice for the Regression Discontinuity Estimator. *Rev. Econ. Stud.* **79**, 933–959 (2012).
2. Uus, A. U. *et al.* BOUNTI: Brain vOlumetry and aUtomated parcellatioN for 3D feTal MRI. *bioRxiv* 2023.04.18.537347 (2023) doi:10.1101/2023.04.18.537347.
3. Studholme, C., Kroenke, C. D. & Dighe, M. Motion corrected MRI differentiates male and female human brain growth trajectories from mid-gestation. *Nat. Commun.* **11**, 3038 (2020).
